# Relative Binding Free Energy between Chemically Distant Compounds Using a Bidirectional Nonequilibrium Approach

**DOI:** 10.1021/acs.jctc.2c00295

**Published:** 2022-06-01

**Authors:** Piero Procacci

**Affiliations:** Dipartimento di Chimica “Ugo Schiff”, Università degli Studi di Firenze, Via della Lastruccia 3, 50019 Sesto Fiorentino, Italy

## Abstract

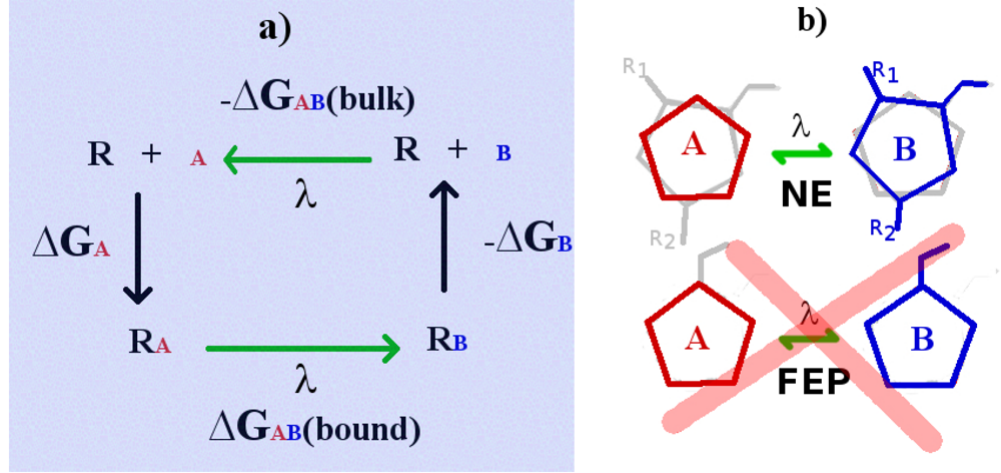

In the context of advanced hit-to-lead drug design based on atomistic molecular dynamics simulations, we propose a dual topology alchemical approach for calculating the relative binding free energy (RBFE) between two chemically distant compounds. The method (termed NE-RBFE) relies on the enhanced sampling of the end-states in bulk and in the bound state via Hamiltonian Replica Exchange, alchemically connected by a series of independent and fast nonequilibrium (NE) simulations. The technique has been implemented in a bidirectional fashion, applying the Crooks theorem to the NE work distributions for RBFE predictions. The dissipation of the NE process, negatively affecting accuracy, has been minimized by introducing a smooth regularization based on shifted electrostatic and Lennard-Jones non bonded potentials. As a challenging testbed, we have applied our method to the calculation of the RBFEs in the recent host–guest SAMPL international contest, featuring a macrocyclic host with guests varying in the net charge, volume, and chemical fingerprints. Closure validation has been successfully verified in cycles involving compounds with disparate Tanimoto coefficients, volume, and net charge. NE-RBFE is specifically tailored for massively parallel facilities and can be used with little or no code modification on most of the popular software packages supporting nonequilibrium alchemical simulations, such as Gromacs, Amber, NAMD, or OpenMM. The proposed methodology bypasses most of the entanglements and limitations of the standard single topology RBFE approach for strictly congeneric series based on free-energy perturbation, such as slowly relaxing cavity water, sampling issues along the alchemical stratification, and the need for highly overlapping molecular fingerprints.

## 1 Introduction

Alchemical free-energy methods based on atomistic molecular dynamics simulations^1−6^ are becoming an important tool in modern drug design, often serving as the last confirmative step for lead identification in the silico drug-discovery process. The impressive rise of computer power provided by high performing computing (HPC) facilities, the consequent drop of the parallel computing cost, and the constant improvement in efficiency, accuracy, and reliability of algorithms and protocols^7−11^ have made in silico screening competitive with the traditional medicinal chemistry practice in the early preclinical stages. The industrial interest in state-of-the-art computer-based drug discovery is testified by the deals, for a total exceeding 100 million dollars, signed by pharmaceutical giants Sanofi, AstraZeneca, Bayer, and BMS in 2015–2020, with Schrödinger, Inc., an American company active in the development of software for computational chemistry.^12,13^

**Figure 1:**
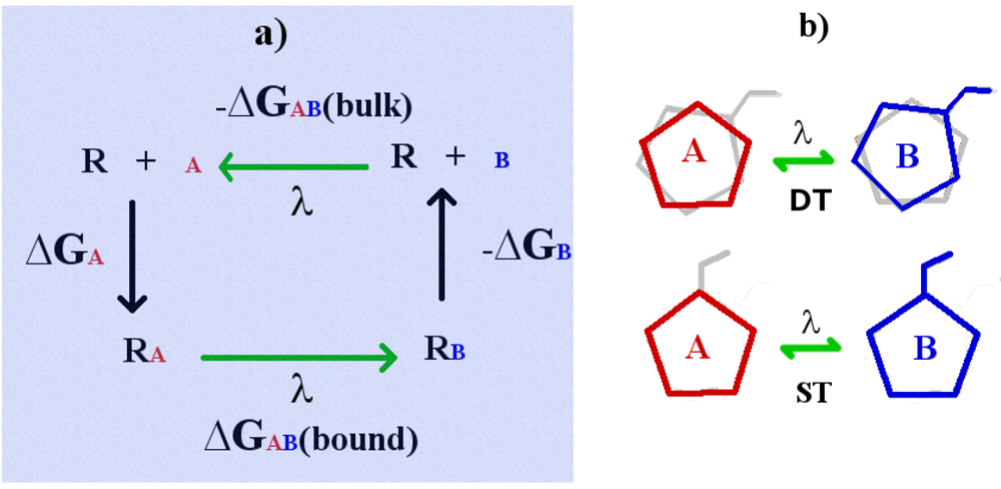
(a) Thermodynamic cycle in RBFE calculations. (b) Dual topology (upper) and single topology (lower) schemes. The gray structure refers to the fully decoupled region.

In industrial settings, MD-based alchemical methods are most often used for ranking the *relative* binding affinities of a series of compounds to a protein target in order to prioritize them for further wet-lab assessement.^14,15^ The relative binding free energy (RBFE) is obtained by computing the free energy cost of transmuting compound A into compound B by way a stratification of nonphysical hybrid λ states (the alchemical path) connecting the two compounds in the bound and unbound arms of a thermodynamic cycle. In Figure 1, the two alchemical transformations in the cycle (bound and bulk states) are indicated by the green arrows, and their free-energy difference equals the difference of the two absolute binding free energies (ABFE), Δ*G*_A_ and Δ*G*_B_. The alchemical cost (Δ*G*_AB_) is generally computed as a sum of free-energy contributions between contiguous λ states using free-energy perturbation (FEP)^16^ or, equivalently, thermodynamic integration (TI),^17^ requiring independent MD simulations for all λ-states (typically from 10 to 20).

The FEP-based or TI-based calculation of the RBFE is much simpler than the direct calculations of the ABFE. The latter requires the setup of a thermodynamic cycle where the ligand is decoupled in the bound state and recoupled in bulk again via λ stratifications. The decoupling process in the bound state is complicated by the tendency of the noninteracting (gas-phase) ligand to drift away from the pocket with the necessity of introducing system-dependent restraints, and by severe sampling issues when the protein switches from the holo form to the apo form.^18^ Nonetheless, in the recent literature, there are few examples of successful FEP-based ABFE calculations in blind SAMPL challenges^19,20^ for symmetrical host–guest systems and (retrospectively) in protein–ligand systems.^21−23^ In the last case, however, the quality of the prediction was found to be strongly dependent on a prior knowledge of the ligand pose.^22^

The current consensus in RBFE calculations is based on the so-called single topology scheme (ST). In this approach (see Figure 1), compound A is transformed to a *strictly congeneric* partner B by interpolating the bonded and nonbonded potential parameters between the two end-states representing A and B. In the most straightforward ST implementation, the two compounds share a common topological structure with the same number of atoms, and a single atom is transformed to another (e.g., H into a halogen atom). When the number of atoms differs as in a so-called “R-group perturbation”^24,25^ (e.g., H into CH_3_ or CH into N in an aromatic cycle), “dummy” atoms are added to the common core structure. While these atoms are fully interacting on one end-state, at the other end, they are bound to the molecule with zero nonbonded parameters, except for 14 nonbonded interactions involving the dummy atoms.^26^ The effect of the dummy atoms should not influence the RBFE as long as this spurious contribution cancels in the two arms of the thermodynamic cycle. Such assumption was recently questioned in ref (24), where the authors showed that the inattentive selection of the bonded terms between dummy and physical atoms may produce errors of the order of *k*_B_*T*, even when evaluating relative hydration free energies between small molecules such as methane and ammonia.

ST-RBFE can be applied to congeneric compounds differing by two or more substituents on a common scaffold. In this case, to avoid the sampling issue related to large transmutation free energies and/or to the enhanced conformational activity of the protein residues upon massive ligand changes, intricate ”perturbation graphs” gradually connecting the interesting molecules must be devised, thereby spending computational resources in determining RBFEs between uninteresting connecting molecules.^27,28^

Single topology RBFE becomes further problematic when dealing with the so-called scaffold hopping transformations involving ring breaking or shrinking, a technique that is widely used in the medicinal chemistry practice.^29^ Recently, many ST schemes have been proposed for RBFE calculations to tackle scaffold hopping.^25,30^ However, the approaches are involved, highly system-dependent, and, as such, not easily amenable to be implemented in automated high-throughput virtual screening for pharmaceutical applications. It should be finally said that the accuracy of ST RBFE calculations has been demonstrated to be dependent on the input poses to construct the perturbation map in congeneric series if sampling is incomplete,^15^ a fact that may further limit the utility of ST RBFE in industrial projects.

The dual topology (DT) scheme is an alternative and conceptually simpler approach in RBFE calculation. In the DT scheme, A and B are present at the same time in both arms of the thermodynamic cycle. In the intermediate λ-states, the two compounds interact with the environment and have no mutual nonbonded interactions, so that they can occupy the same volume while the simultaneous alchemical transformations of A into B and B into A occur. Apparently, DT might be considered the most straightforward approach when the two end-state molecules are chemically distant. However, as noted in ref (31), a dual-topology calculation is, in essence, the same as two absolute free-energy calculations where coupling (creation), and decoupling (annihilation) are executed simultaneously in opposite directions. Hence, DT schemes are believed to suffer from the same pathologies exhibited by FEP-based ABFE calculations, with difficulties in sampling and slow convergence.^31^

The DT scheme has been revived in two recent papers.^31,32^ In ref (32), Gallicchio and co-workers proposed an extension for RBFE of their FEP-based alchemical transfer method (ATM)^33^ for ABFE, a two-arm alchemical technology where the common intermediate state is a *mixture* of the solvated bound and unbound complex instead of vacuum as in standard FEP for ABFE. In the ATM-RBFE variant, the two end-states are the A-bound complex and B in bulk and vice versa, while the common intermediate state is an AB alchemical mixture in the bound state *and* in bulk. In the work by Roux and co-workers,^31^ the alchemical end-states in the cycle of Figure 1 contain both A and B, one fully coupled and the other decoupled, sharing a common substructure where each pair of corresponding atoms of A and B is holonomically constrained to share similar coordinates at all time during the λ-alchemical simulations. Both technologies are based on FEP and, in principle, could be used to evaluate RBFE for scaffold hopping transformations. In practice, these ingenious DT methods, requiring convergence on all intermediate λ states, still face severe sampling issues when the alchemical part of the hybrid A+B molecule is large. Both techniques have been either applied to strictly congeneric compounds^31^ or tested on structurally similar compounds imposing an appropriate restraining potential that maintains the ligand alignment in order to enhance the convergence of the calculations.^32^

In this paper, we propose a straightforward dual topology alchemical approach for calculating the binding free energy between two arbitrary chemical compounds, with disparate connectivity, volume, and net charge. The method relies on the enhanced sampling of the A(B) and B(A) end-states in bulk and in the bound state, alchemically connected by a series of independent fast nonequilibrium (NE) simulations affording the calculation of a NE work distribution. Enhanced sampling is performed using an efficient solute tempering Hamiltonian Replica exchange scheme (ST-HREM)^34^ and is required *only for the true physical states* corresponding to the solvated complex and the compound in bulk. The hybrid dual topology starting end-states are generated by combining gas-phase configurations of the ghost molecule with the ST-HREM sampling of the true end-states. The method constitutes an extension of our NE alchemy for ABFE calculation termed virtual double system single box (vDSSB),^35−38^ and, unlike the ABFE variant (unidirectional by design^38^), can be also implemented in a bidirectional fashion, applying the Crooks theorem to the forward and reverse work distribution and exploiting the accuracy and precision of the Bennett Acceptance Ratio.^39^ In this paper, we have used our approach for RBFE on the important test bed provided by the recent SAMPL9 challenge,^40^ involving the binding affinity of a series of noncongeneric compounds for the WP6 macrocyclic host.^41^

## 2 Theoretical Background

NE alchemy for RBFE is implemented using the thermodynamic cycle of Figure 1 as in standard FEP alchemy, but replacing the intermediate λ-states simulations with a swarm of fast NE alchemical transitions. This methodology was recently applied^42^ to a standard benchmark dataset for relative binding free energy of strictly congeneric series.^10,11^ NE alchemy was systematically compared to ST-FEP and ST-FEP+, which is an FEP variant implemented in the proprietary code Desmond^7^ featuring the enhanced sampling of the binding pocket in the intermediate states of the alchemical path.^10,43^ It was found that^42^ “the non-equilibrium free energy calculations performed comparably to FEP+ and reached a mean unsigned error of 3.70 kJ mol^–1^”. In ref (42), NE alchemy was applied in a rather conservative fashion, strictly retracing the standard ST FEP-based alchemical approach for congeneric ligands with R-group perturbations. Basically, the λ-states equilibrium simulations in FEP were substituted by a few hundreds of NE alchemical simulations in both directions, each lasting 50 ps, recovering the RBFE from the crossing point of the forward and reverse work distributions via the Bennett Acceptance Ratio.^44^ No enhanced sampling of the end-states was performed, nor was an attempt made to check whether the technology could be extended to compounds not sharing a large common core structure.

Here, as in ref (42), we use a swarm of independent NE simulations to perform the alchemical connection from A to B, recovering the forward *P*_AB_(*W*_b/u_) and time-reversal backward *P*_BA_(−*W*_b/u_) work distributions. At variance with ref (42), we use the DT scheme, with the forward (eq 1) and reverse (eq 2) transformations being defined as
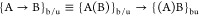
1
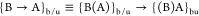
2In this notation, (A), (B) denotes the decoupled ghost, while the superscripts b or u indicate that the alchemical NE process is conducted in the bound and unbound state, respectively. The sampling of the four end-states of Figure 1, {A(B)}_b*/*u_, {B(A)}_b*/*u_, are obtained using replicates of Hamiltonian Replica Exchange with solute tempering^45^ with only *intrasolute* scaling^34^ along the replica progression (i.e., leaving the solvent cold), hence affording an intrasolute temperature of thousands of Kelvin, using a limited number of replicas.

During the alchemical simulations, the ghost-environment interaction potential is gradually switched on, while that of the partner is simultaneously switched off. In the NE unbound transition, the two molecules *do not sense each other* and are characterized by the full *intramolecule* potential. In the bound NE transitions, the two molecules do not sense each other but their centers of mass (COM) are tethered via a simple harmonic potential of the form *V*_rstr_ = ^1^/_2_*K*(Δ*R*)^2^, with Δ*R* being the COM–COM distance and with the force constant *K* set to 2 kcal mol^–1^ Å^–2^. The COM–COM weak restraint between the two ligands does not affect, in any way, their relative conformations during the transitions, as the two alchemical molecules do not interact with each other, and serves only to keep the growing and annihilating molecules in the binding pocket at all times. The COM–COM restraint may have a limited impact on the *dissipation* of the process (defined as the difference between the mean work and the underlying free energy) but not on the final free energy values between the thermodynamic end-states (a rigorous proof is given in the Appendix). The latter can be recovered exploiting the Crooks theorem on the forward *and* reverse work distributions, or using the Jarzynski identity^46^ on the forward *or* reverse distributions. Note that, if, in the alchemical process, the net charge of the system changes, then a finite size correction must be applied a posteriori to the RBFE. In NE-RBFE calculations, these finite-size corrections are trivially given by the difference of the finite-size corrections of the corresponding ABFE′ (see eq 9 of ref (47)).

The strength of NE alchemy for RBFE, compared to FEP, lies in the fact that the need for canonical sampling in the intermediate states of the alchemical path is totally bypassed. Accurate sampling is needed only for the physical end states involving one molecule, with the dual topology initial configurations being trivially obtained by combining these physical states with gas-phase configurations of the ghost partner. The computational paradigm is shifted from *single molecule* time averages to a corresponding *ensemble* averages derived from time-unordered canonical configurations sampled with replicates of HREM simulations. The end-state canonical distribution of the initial equilibrium state is reflected in the resulting work distribution obtained in the driven alchemical transitions to the final nonequilibrium end-state. If, for example, a molecule can bind with two possible metastable poses with different free energies separated by large energy barriers, then the work distribution where this molecule is rapidly annihilated and the other is created is expected to exhibit a corresponding bimodal character.

The work distribution can hence be seen as the fingerprint of the initial canonical sampling, *blurred* by the dissipation of the process defined as *W*_diss_ = ⟨*W*⟩ – Δ*G*. The latter grows linearly with the speed^48^ of the process and is noticeably dependent on the selected alchemical protocol. In our case, provided that the molecules have similar volumes, thanks to the COM–COM restraint, the growth process occurs with little resistance (i.e., small dissipation) in an “empty” region occupied by the (unperceived) annihilating partner. Therefore, NE-RBFE *is not equivalent* to two NE-ABFE calculations. The variance or dissipation in the DT NE-alchemical process is generally much less than the sum of the variance of the annihilation and growth processes combined (see Figure S19 in the SI). When the volume of the two molecules is disparate, steric clashes while a molecule is growing can dramatically enhance the NE work in most of the NE trajectories, negatively affecting the accuracy and precision of the free-energy estimate. To further limit the dissipation in the early stage of the growth process, we use a modification of the soft-core regularization proposed in ref (49), based on shifted, rather than softened, electrostatic and Lennard-Jones potential of the form
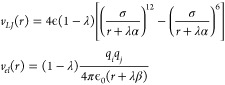
3where the shifted origins α = 0.35 Å and β = 4.0 Å have been tuned to minimize the dissipation. On the Zenodo public repository https://zenodo.org/record/6385017, we provide two small movies that illustrate the evolution of the Beutler soft-core^49^ potential and of the shifted potential of eq 3, from λ = 1, corresponding to the ghost molecule, to λ = 0 corresponding to the full interacting compound.

## 3 Methods

### 3.1 Simulation Setup and Parameters

**Figure 2:**
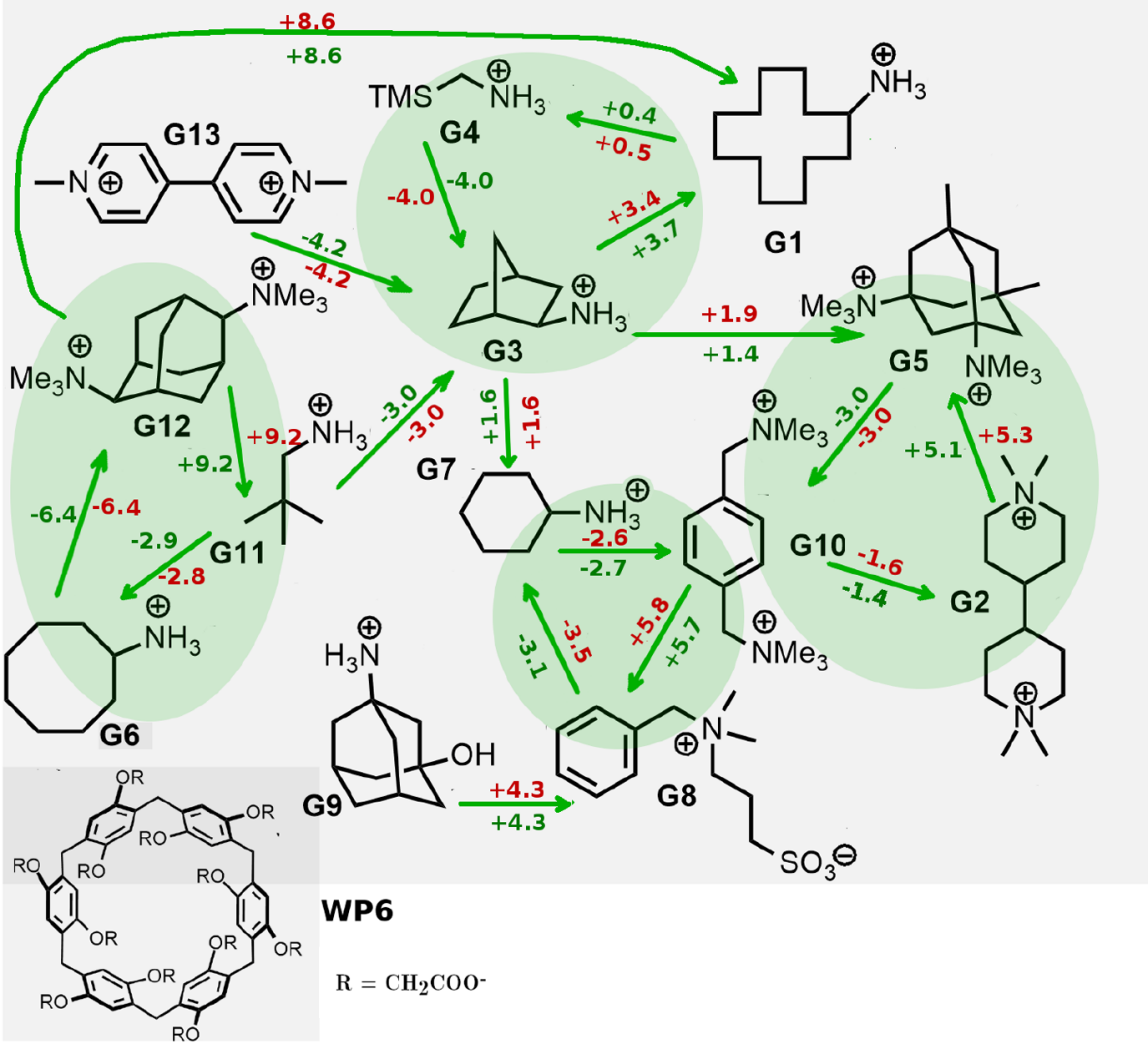
Guests (Gn) and host (WP6) of the SAMPL9 challenge. The green arrows indicate the pairs for which we computed the RBFEs. The latter (in kcal/mol) are reported in green and red for the BAR and BAR* estimates, respectively (see below). Primary cycles for cycle closure assessment are shown in the highlighted green region.

Guests and host PDB files were taken from the SAMPL9 GitHub site.^40^ The protonation state of all species is indicated in Figure 2. For the two chiral compounds, the tested G3 guest (2-bicyclo[2.2.1]heptanyl]azanium) corresponds to the 1R 2R 4S diastereoisomer. The tested G12 guest (hexamethyladamantane-2,6-diammonium) has 2 and 6 carbon atoms of the R type. The force field (FF) parameters and topology of the host and guests molecules were prepared using the PrimaDORAC interface,^50^ based on the GAFF2^51^ parameter set. For G4, the silicon-related parameters were taken from ref (52). The initial bound state was prepared using the Autodock Vina code.^53^ The bound complexes and the ghost ligands were solvated in ∼1600 and 512 OPC3^54^ water molecules, respectively. Long-range electrostatic interactions were treated using the Smooth Particle Mesh Ewald (SPME) method.^55^ A background neutralizing plasma was assumed within the SPME method.^56^ The cutoff of the Lennard-Jones interactions was set to 13 Å. All simulations, HREM or nonequilibrium, were performed in the NPT ensemble under standard conditions, using an isotropic Parrinello–Rahman Lagrangian^57^ and a series of Nosé thermostats^58^ for pressure and temperature control, respectively. Bond constraints were imposed on X–H bonds only, where X is a heavy atom. All other bonds were assumed to be flexible. All simulations have been done using the hybrid OpenMP-MPI program ORAC^59^ on the CRESCO6 cluster.^60^

### 3.2 HREM Simulations

HREM simulations have been performed for the 13 complexes Gx-WP6 and the 13 solvated ligand, for a total of 26 HREM runs. HREM uses *n* = 16 and *n* = 12 replicas for the bound and unbound states, respectively, with a maximum scaling factor of *S* = 0.1 (corresponding to a temperature of 3000 K) involving only the intrasolute bonded and nonbonded interactions.^34^ The scaling factors along the replica progression are computed according to the protocol *S*_*m*_ = *S*^(*m*–1)/*n*^ with *m* = 1, ..., *n*. HREM simulations were performed in a single parallel job, using the ORAC option BATTERY,^59,61^ in eight replicates, each lasting 6 ns for the bound ligand and 2 ns for the ligand in bulk, for a total of 48 and 12 ns for the bound and unbound state, respectively. The exchange rates were in the range of 25%–50%. The HREM jobs for the bound state engaged 128 MPI processes, each using 6 OpenMP threads for a total of 768 cores. The jobs were completed within ∼8 wall clock hours on the CRESCO6^60^ cluster. For the unbound state, we used 96 MPI processes with 6 threads each, for a total of 576 cores, taking a wall clock time of 1 h. On the Zenodo platform (https://zenodo.org/record/6385017), we provide the HREM-generated trajectories of the bound and unbound state for all 13 guest–host systems.

### 3.3 Preparation of the Initial States for the NE Dual Topology Runs

The dual topology end-states for the bound systems, {A(B)}_b_ and {B(A)}_b_, are prepared by combining 196 phase-space points of the solvated complex taken from the 48 ns HREM, with an equivalent number of gas-phase configurations of the ghost molecule also sampled with HREM in a separate simulation (8 ns of the isolated molecule) and performed on a low-end workstation within <5 min. In each starting configuration for the bound state, the COM of the ghost molecule is made coincident with the COM of the fully coupled partner. The dual topology end-states for the ligand in bulk, {A(B)}_u_ and {B(A)}_u_ are prepared by combining 96 gas-phase configurations of the ghost molecules with 96 snapshots taken at regular intervals from the 16 ns HREM simulation of the fully coupled compound in bulk solvent.

### 3.4 NE-Alchemy Runs

Each of 196 NE-alchemy DT transitions for the complex lasted 0.72 ns with identical and simultaneous linear protocols for Lennard-Jones and electrostatic interactions and were run in both directions (i.e., {A(B)}_b_ → {(A)B}_b_ and {B(A)}_b_ → {(B)A}_b_. During the alchemical transition, the COM–COM harmonic potential helps to maintain both molecules in the binding site (see section 2). For the unbound arm, we produced 96 NE-alchemy DT runs, each lasting 0.36 ns in the forward and reverse direction. Therefore, for each couple AB, we run 392 NE-alchemical trajectories in the bound state, for a total simulation time of ∼280 ns and 192 trajectories in bulk for a total time of ∼70 ns. A parallel job for the bound state in one direction uses 1176 cores (196 MPI × 6 threads) delivering the work file within <2 wall clock hours on CRESCO6. For the unbound state, the job in one direction uses 576 cores (96 MPI× 6 threads) for 20 wall clock minutes. The computational demand of BAR-based (bidirectional) NE-RBFE is similar to that that of our ABFE SAMPL9 submisson,^47^ where we used ∼400 annihilation (unidirectional) NE trajectories 1.44 ns long in the bound state.

## 4 Results

In Figure 2, we show the 13 guests and the WP6 host of the SAMPL9 challenge. In total, we have computed 18 RBFE, as indicated by the green arrows in Figure 2. In Table 1, for each pair, we report the Tanimoto coefficient^62^ (computed using the PubChem fingerprints^63^), the volume and charge change, and the change in the number of rings.

**Table 1:** Tanimoto Coefficient (*T*), Volume Change (Δ*V*), Charge Change (Δ*Q*), and Change in the Number of Rings (Δ*n*_rings_) in the RBFE Calculations

pair	*T*	Δ*V*(Å^3^)	Δ*Q*(*e*)	Δ*n*_rings_
g03 → g01	0.55	72.01	0	–1
g04 → g01	0.21	76.98	0	+1
g12 → g01	0.44	–39.90	–1	–3
g05 → g02	0.53	–41.91	0	–2
g10 → g02	0.37	12.58	0	+1
g04 → g03	0.15	4.97	0	+2
g05 → g03	0.38	–145.55	–1	–2
g07 → g03	0.61	4.29	0	+1
g11 → g03	0.40	9.54	0	+2
g13 → g03	0.17	–35.63	–1	0
g10 → g05	0.34	54.48	–1	+3
g11 → g06	0.50	30.97	0	+1
g12 → g06	0.43	–90.48	–1	–3
g08 → g07	0.21	–77.36	+1	0
g10 → g07	0.24	–95.36	–1	0
g09 → g08	0.22	35.17	–1	–3
g10 → g08	0.80	–18.00	–2	0
g12 → g11	0.29	–121.45	–1	–4

In typical ST-RBFE perturbations for isocharged congeneric series,^10,64^ the mean Tanimoto coefficient is in the range of 0.85–0.98, and the volume change involves, in most cases, the volume of one single substituent (e.g., H → CH_3_). In this study, as can be seen from Table 1, each transformation is characterized, in most cases, by a Tanimoto coefficient *T* of <0.5.^65^ When *T* > 0.5, as in g10 → g08 and g07 → g03, the transformation involves a charge change of 2*e* and a variation of the number of rings, respectively. Many of the transformations are characterized by at least one index of chemical dissimilarity, as measured by *T*, Δ*V*, and Δ*n*_rings_.

### 4.1 Bidirectional and Unidirectional NE-RBFE Estimates

In Table 2, we report various estimates of the RBFE for the 18 pairs. The notation gx → gy signifies that the corresponding RBFE refers to the transmutation of gx into gy, with the DT scheme indicated as gx(gy) → (gx)gy. The quantity δ*G*_fs_ refers to the finite size correction under periodic boundary conditions and PME^56^ when the transmutation involves two ligands with different charges. The finite charge corrections for the absolute binding free energy of the 13 guests are reported in ref (47). The raw values of the work for all the forward and reverse transformations involving the 18 pairs are provided on the Zenodo public repository (https://zenodo.org/record/6385017), along with a simple bash scripts for processing the raw data to yield bidirectional (BAR-based) or unidirectional (Jarzynski or Gaussian) estimates.

**Table 2:** Results of Free Energy Calculations for the AB Trasmutation for 18 Pairs of the SAMPL9 Challenges^a^

pair	BAR	BAR*^b^	δ*G*_fs_	*J*(FF)	*J*(RR)	J*(FR)	J*(FR)	*G*(FF)	*G*(RR)
g03 → g01	3.7 ± 0.6	3.4	0.0	2.6 ± 0.8	3.8 ± 1.6	2.0 ± 0.7	4.3 ± 1.3	0.5 ± 2.0	6.5 ± 2.0
g04 → g01	–0.4 ± 0.6	–0.5	0.0	–0.2 ± 0.7	–0.5 ± 1.1	–0.6 ± 1.0	–0.1 ± 0.7	–3.2 ± 2.0	3.3 ± 1.6
g12 → g01	8.6 ± 0.9	8.6	–2.3	9.0 ± 0.9	8.7 ± 1.2	8.8 ± 0.8	8.6 ± 1.2	3.7 ± 3.5	9.6 ± 1.9
g05 → g02	–5.1 ± 0.4	–5.3	0.0	–3.0 ± 0.4	–7.2 ± 0.8	–3.2 ± 0.6	–7.2 ± 0.7	–4.2 ± 0.8	–
g10 → g02	–1.4 ± 0.6	–1.6	0.0	–0.2 ± 1.3	–3.6 ± 1.0	0.3 ± 1.6	–4.2 ± 0.6	–2.8 ± 2.1	–0.2 ± 1.6
g04 → g03	–4.0 ± 0.3	–4.0	0.0	–4.0 ± 1.0	–5.0 ± 0.7	–4.3 ± 0.5	–5.0 ± 0.5	–5.6 ± 1.0	–4.1 ± 0.6
g05 → g03	–1.3 ± 0.7	–1.3	–2.3	1.3 ± 0.7	–2.2 ± 3.3	1.5 ± 0.7	–2.2 ± 2.7	0.9 ± 0.7	–
g07 → g03	–1.6 ± 0.3	–1.6	0.0	–2.6 ± 2.2	–2.1 ± 0.5	–2.5 ± 1.8	–2.7 ± 0.5	–3.3 ± 1.2	–0.7 ± 0.8
g11 → g03	–2.9 ± 0.3	–2.9	0.0	–2.3 ± 0.7	–3.7 ± 1.0	–2.6 ± 0.6	–3.6 ± 0.8	–3.7 ± 1.0	–3.0 ± 0.7
g13 → g03	–4.2 ± 0.4	–4.2	–2.3	–3.0 ± 0.6	–5.3 ± 0.8	–2.8 ± 0.8	–5.5 ± 0.6	–7.9 ± 1.5	–3.9 ± 1.2
g10 → g05	3.0 ± 0.5	3.0	0.0	3.5 ± 2.0	2.0 ± 1.3	3.6 ± 2.2	1.9 ± 1.2	–	2.4 ± 0.8
g11 → g06	–2.9 ± 0.3	–2.8	0.0	–1.8 ± 1.0	–3.2 ± 1.2	–1.6 ± 1.1	–3.1 ± 1.0	–3.9 ± 1.1	–2.5 ± 1.1
g12 → g06	6.4 ± 0.3	6.4	–2.3	7.7 ± 0.5	5.8 ± 0.9	7.9 ± 0.4	5.6 ± 1.1	5.8 ± 0.9	7.6 ± 1.2
g08 → g07	–3.1 ± 0.8	–3.5	2.0	–3.4 ± 2.7	–3.6 ± 1.3	–1.2 ± 2.2	–6.0 ± 1.8	–4.7 ± 2.1	2.9 ± 3.4
g10 → g07	2.7 ± 0.4	2.6	–2.3	4.8 ± 0.9	0.0 ± 0.6	4.8 ± 1.1	0.0 ± 0.4	3.2 ± 1.1	4.6 ± 1.6
g09 → g08	4.3 ± 0.8	4.3	–2.0	4.6 ± 1.7	3.8 ± 1.2	6.2 ± 1.1	1.5 ± 2.1	2.1 ± 2.0	6.9 ± 2.0
g10 → g08	5.7 ± 0.9	5.8	–4.3	7.2 ± 1.2	4.6 ± 2.1	8.3 ± 1.4	3.2 ± 2.0	4.4 ± 1.7	6.4 ± 2.3
g12 → g11	9.2 ± 0.4	9.2	–2.3	10.2 ± 0.6	9.2 ± 0.8	10.1 ± 0.6	9.3 ± 0.7	9.0 ± 0.9	11.0 ± 1.7

a Legend: BAR, Bennett acceptance ratio bidirectional estimate; BAR*, Bennett acceptance ratio bidirectional estimate using vDSSB; *δG*_fs_, finite size correction due to charge change (see text); J(FF), Jarzynski forward AB process; J(RR), Jarzynski reverse BA process; J*(FR), vDSSB estimate with convolution of bound forward and unbound reverse distributions; J*(RF), vDSSB estimate with unbound forward and bound reverse distributions; G(FF), Gaussian estimate forward; and G(RR), Gaussian estimate reverse. All estimates are in kcal/mol. Reported errors have been computed by bootstrap with resampling.

b BAR* errors (not reported) are <0.1 kcal/mol in all cases. *Nota bene*: all reported estimates include δ*G*_fs_.

BAR and BAR* are the two possible *bidirectional* estimates with the proposed approach. ΔΔ*G*_BAR_ corresponds to the difference of the bound and unbound arm perturbations (see Figure 1), each computed using BAR on the forward and reverse transformation. ΔΔ*G*_BAR*_ is obtained by applying the BAR to the *convolution* of the forward and reverse vDSSB processes, i.e.,
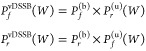
4

**Figure 3:**
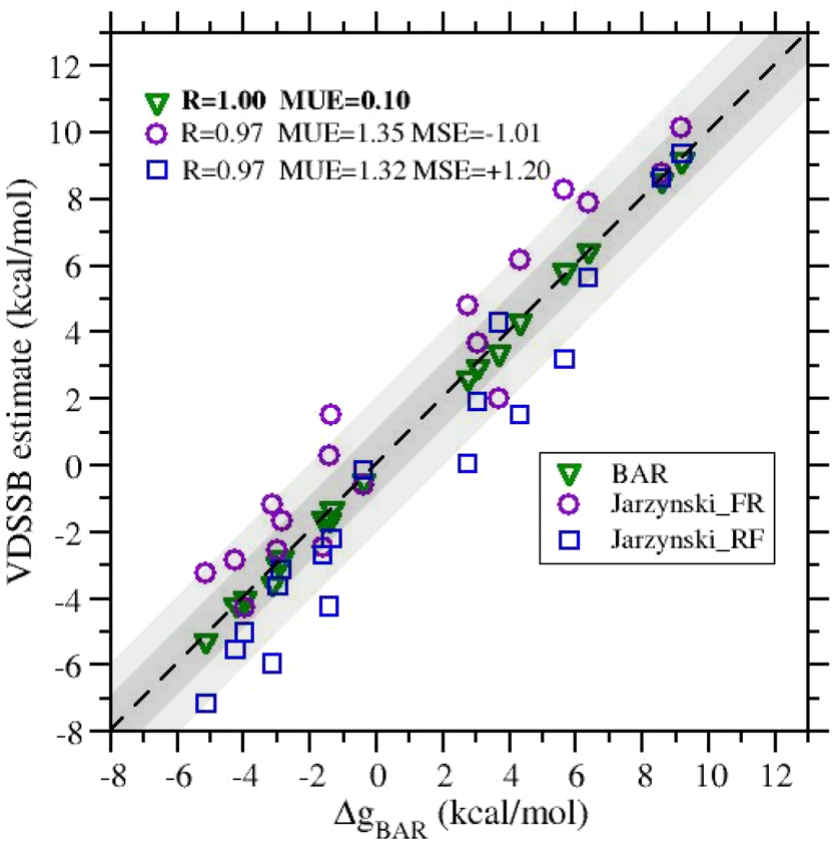
vDSSB unidirectional and bidirectional estimates of the RBFE (see text) vs the BAR estimate.

J*(FR) and J*(RF) are unidirectional Jarzynski estimates based on the convolution of the work distribution of the forward bound process and the reverse unbound process and vice versa. *G*(FF) and *G*(RR) are unidirectional Gaussian estimated, computed separately on the two arms of the thermodynamic cycle. For the transformations involving g05, where this compound starts as a ghost, the distribution is markedly non-normal (see work distributions involving G5 in the SI) and no Gaussian estimate is reported. In the correlation plot of Figure 3, the two-arms standard BAR estimate is compared to the vDSSB-based bidirectional (BAR*) and unidirectional (J*(FR) and J*(RF)) estimates. Basically, BAR and BAR* are coincident.

**Figure 4:**
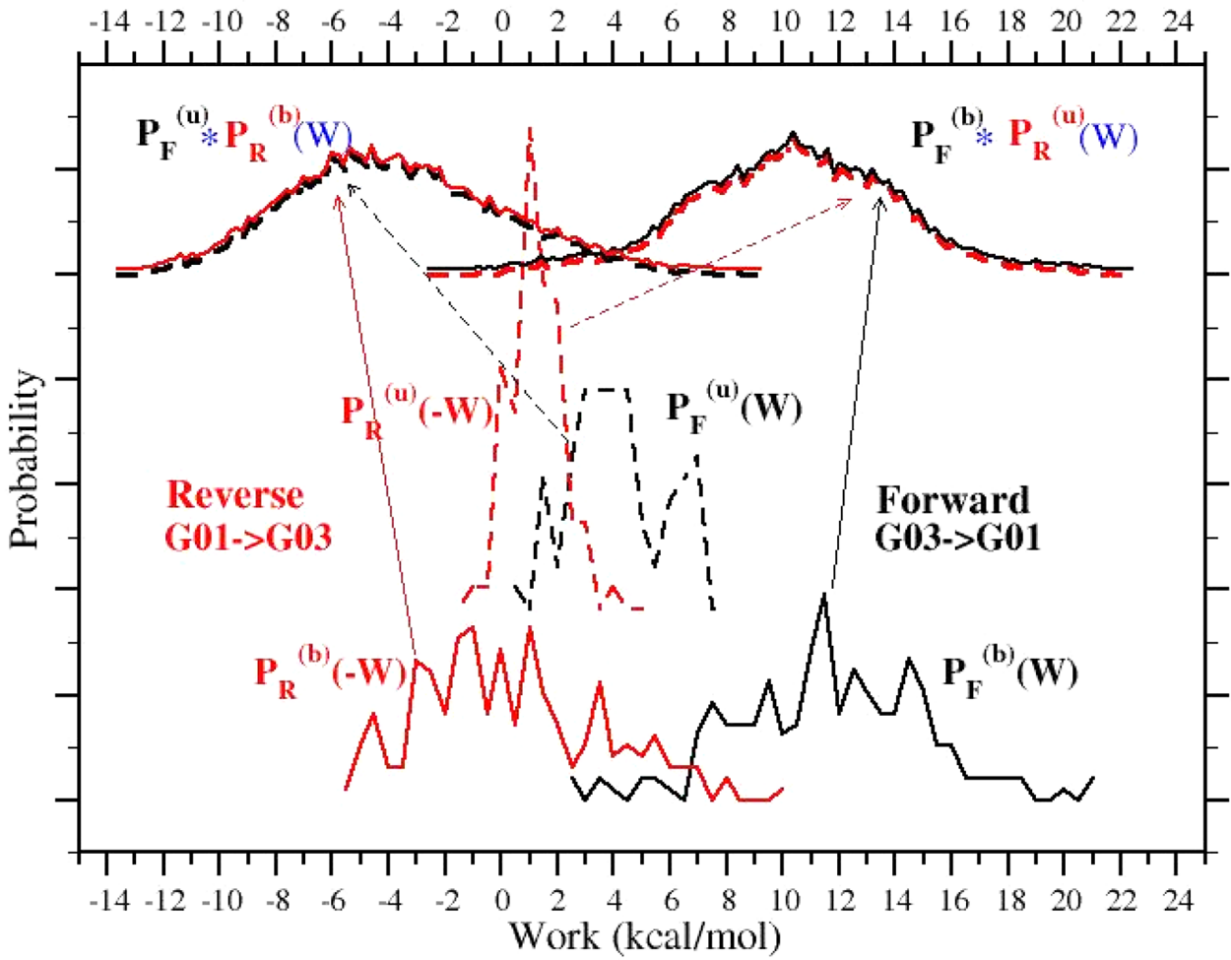
Work distributions in the G03 → G01 transformation.

Both these estimates involve *four* transitions—two in the bound state and two in the unbound state—but they are not equivalent, as the overlap of the forward and reverse distribution in the convolution is smaller than that of the individual bound and unbound distribution, as can be seen in the example reported in Figure 4.

The bound state forward work *W*_*f*_^(b)^ and the unbound state reverse work *W*_*r*_^(u)^ are two independent random variables. Hence, the mean value of their sum is given by the sum of the individual mean values, i.e., *W̅*_*f*_^vDSSB^ = *W̅*_*f*_^(b)^ + *W̅*_*r*_^(u)^ and *W̅*_*r*_^vDSSB^ = *W̅*_*r*_^(b)^ + *W̅*_*f*_^(u)^ and the variances are σ_*f*_^2^(vDSSB) = σ_*f*_^2^(b) + σ_*r*_^2^(u) and σ_*r*_^2^(vDSSB) = σ_*r*_^2^(b) + σ_*f*_^2^(u). Therefore, the corresponding convolution distributions *P*_*f*_^vDSSB^(*W*) and *P*_*r*_^vDSSB^(−*W*) have a tendency to widen and spread apart, reducing the overlap. However, accuracy is preserved, since the convolution is much more resolved than the individual distributions on the right-hand side (rhs) of eq 4. These facts are illustrated in Figure 4.

**Figure 5:**
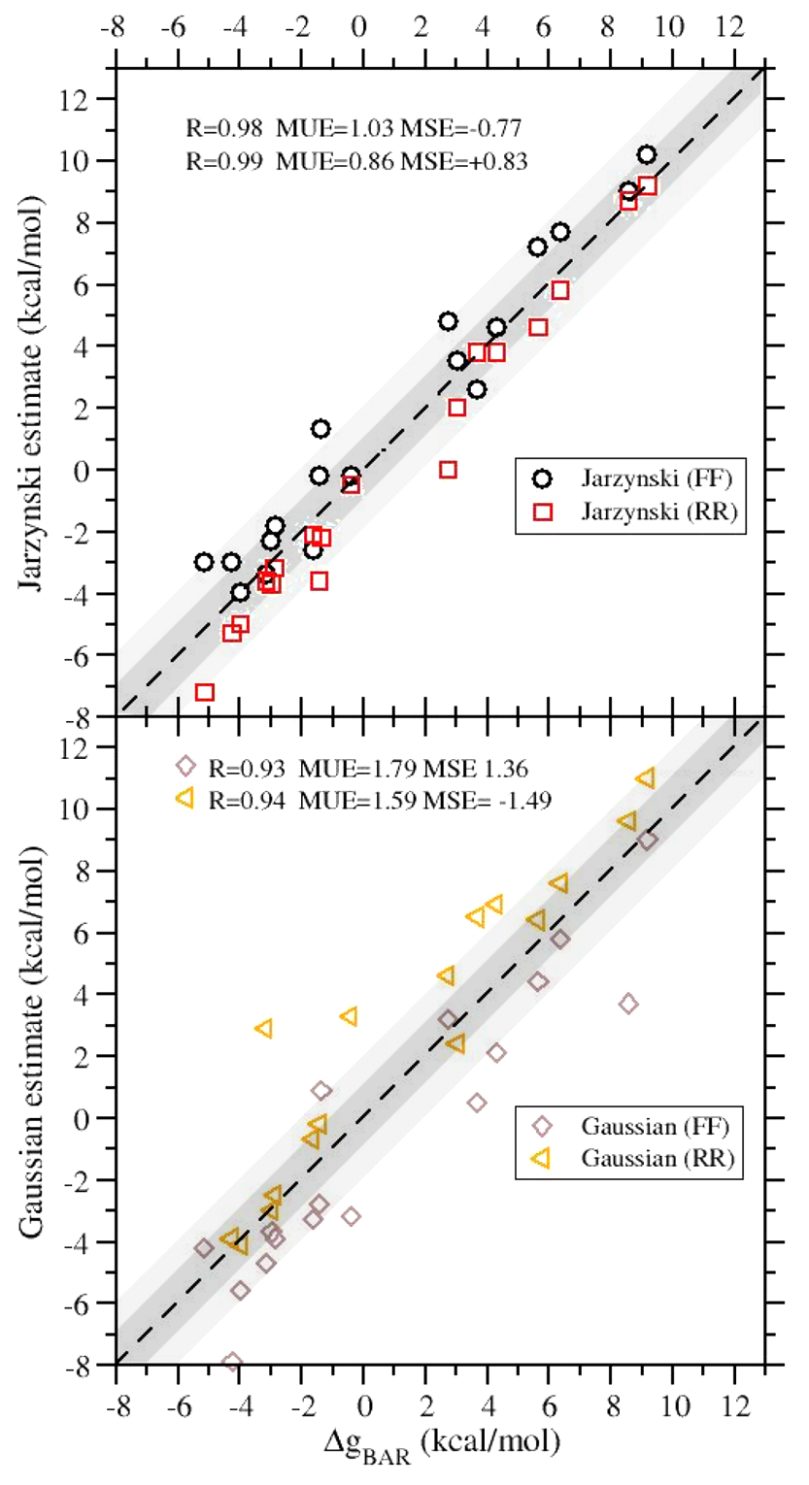
(Upper panel) Correlation diagram between the BAR and *J*(FF) and *J*(RR) estimates. (Lower panel) Correlation diagram between the BAR and *G*(FF) and *G*(RR) estimates (see text).

BAR is notoriously the most accurate estimator in free-energy calculations. However, assessment of the unidirectional estimates is important, since these allow one to spare half of the computational time for NE alchemical stage. In this regard, J*(FR) does not coincide with J*(RF) as the dissipation can be different in the two senses (again, see Figure 4). Generally, we note that J*(FR) and J*(RF) are overestimated and underestimated, with respect to the BAR estimates, respectively. By the same token, *J*(FF) and *J*(RR) (see Figure 5) generally appear to overestimate and underestimate the BAR RBFE, respectively. The situation is reversed for the estimate G(FF) and G(RR) with the former being systematically underestimated, with respect to BAR. Evidently, our choice of the 18 transitions of Figure 2 and the (arbitrary) assignment of their forward sense (see Table 2) must have had a systematic impact on the unidirectional estimates.

In Table 3, we have computed the Pearson correlation coefficient of the deviation from the BAR estimate of each of the unidirectional estimates of Table 2 with the corresponding metrics for chemical dissimilarity given by the relative charge change (*C*_q_ = , the relative volume change (*C*_v_ = , and the Tanimoto coefficient *T* in the 18 alchemical transformations. The Tanimoto coefficient does not appear to be correlated to the observed discrepancies between bidirectional (BAR) estimate and unidirectional estimate. Some significant correlation is observed for the charge and for the volume change. Remarkably, for the latter, a moderate negative and weakly positive or null correlation is observed in the forward and reverse process, respectively. Stated in other terms, when the volume of the B ligand is *much less* than the volume of the A ligand, the unidirectional estimates has a tendency to deviate from the BAR estimate with a systematic sign. In the reverse direction, the correlation is significantly weaker, but the systematic deviation still remains. These data seem to suggest that volume and charge dissimilarity in the AB transition, rather than chemical fingerprints dissimilarity, are more likely to affect the accuracy of unidirectional estimates.

**Table 3:** Pearson’s Correlation Coefficient of the Deviation between Unidirectional and Bidirectional Estimates with the Charge, Tanimoto, and Volume Changes^a^ in the 18 Transformations of Table 2

error	charge change, *R*(*C*_*q*_)	change in Tanimoto coefficient, *R*(*T*)	volume change, *R*(*C*_*v*_)	MSE (kcal/mol)
J*(FR)-BAR	0.57	–0.03	–0.60	1.01
J*(RF)-BAR	–0.54	0.08	0.24	–1.20
*J*(FF)-BAR	0.10	0.03	–0.57	0.77
*J*(RR)-BAR	0.09	0.07	0.24	–0.83
*G*(FF)-BAR	–0.04	0.10	–0.52	–1.36
*G*(RR)-BAR	0.47	–0.25	–0.13	1.49

a See text.

We conclude this section by comparing the results with vDSSB^47^ for ABFE with the BAR-based RBFE reported in Table 2. vDSSB-ABFE are *unidirectional* estimates relying on the convolution of the bound state annihilation work distribution with the unbound state growth work histogram. The ABFE for the 13 guests of SAMPL9 are tabulated as the first entry in Table 2 in ref (47). They include the volume correction due to the receptor–ligand restraint (eq 8 in ref (47)) and the so-called finite-size correction (FSC)^56^ that applies to charged ligands (eq 9 in ref (47)).

**Figure 6:**
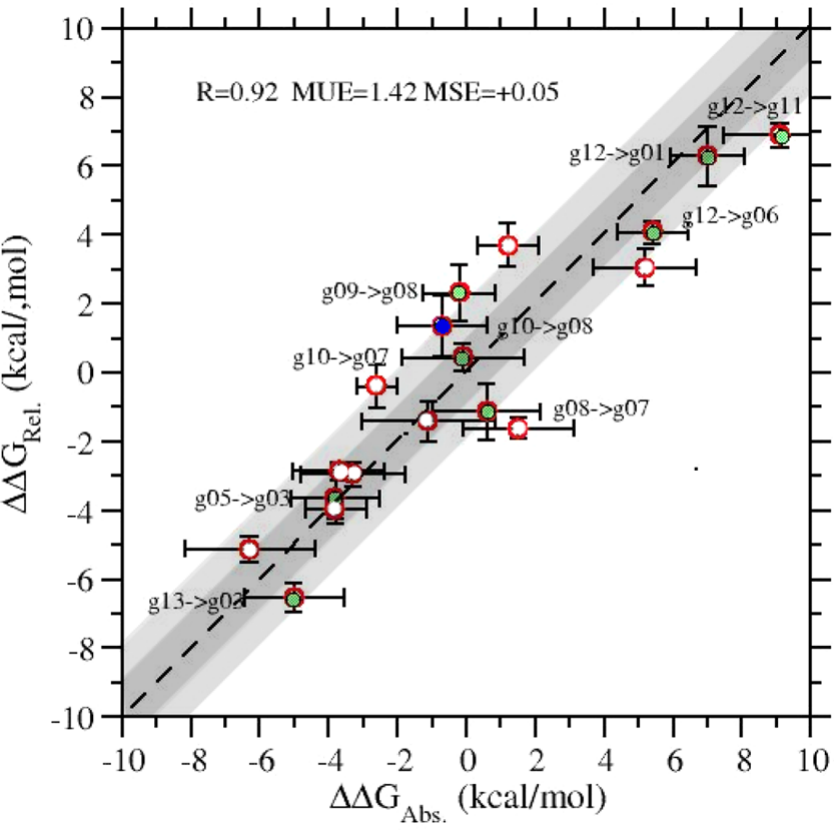
ABFE/RBFE correlation diagrams for the 18 transmutations of Table 2. White, green, and blue symbols indicate a charge change in the transmutation of 0, 1, and 2 electrons.

For RBFE, while volume correction does not apply, FSC must be accounted for when, in the transmutation, the net charge of the ligand varies. Values of the relative FSC for all 18 transitions are reported in Table 2. The ABFE/RBFE correlation diagram is shown in Figure 6. ABFE and RBFE are strongly correlated (*R* = 0.98) exhibiting a mean unsigned error (MUE) of 1.5 kcal/mol, with most of the RBFE data lying in the 2 kcal/mol error region and within the ABFE error. In this regard, note that the mean error in the BAR-based RBFE is much less than that of the Jarzynski-based error of the ABFE. There is apparently no systematic bias between ABFE and RBFE, as testified by the negligible mean signed error (MSE) and by a best-fitting line with *a* ≈ 0.9 and *b* ≈ 0. Figure 6 represents a strong mutual validation of the two NE-based techniques for ABFE^38,47^ and RBFE calculation.

### 4.2 Cycle Closure Conditions

The so-called cycle closure condition^32,64^ (CCC) represents a very stringent test for assessing the reliability and precision of RBFE calculations. The free-energy change in a cycle, regardless of the number of edges, should be zero. In Figure 2, the four primary three-edge cycles are highlighted in green. In two of these cycles (g01 → g03 → g04 → g01 and g10 → g02 → g05 → g10), the alchemical transmutations involve isocharged ligands with one and two net charges. In the other two cycles (g06 → g12 → g11 → g06 and g07 → g10 → g08 → g07), the ligand net charge may change by one or two electron units. Besides, CCC can be tested also on secondary cycles involving more than three edges. In Table 4, CCC has been tested using the BAR and BAR* bidirectional estimates for all possible primary (3 edges) and secondary cycles (4, 5, 6 edges) in the RBFE network of Figure 2. CCC is satisfied for the three-edge cycles with errors largely within the edge confidence intervals summed in quadrature. Remarkably CCC is still satisfied within the confidence interval in cycles up to six edges, irrespective of changes in chemical similarity, ligand charge, volume or number, and nature of rings.

**Table 4:** Cycles Closure Conditions in the Network of Figure 2

cycle	BAR (kcal/mol)	BAR* (kcal/mol)
g01 → g03 → g04 → g01	–0.1 ± 1.2	0.1
g10 → g02 → g05 → g10	0.7 ± 1.2	0.6
g06 → g12 → g11 → g06	0.0 ± 1.0	0.0
g07 → g10 → g08 → g07	–0.4 ± 1.4	–0.3
g12 → g01 → g03 → g11 → g12	1.3 ± 1.5	1.1
g03 → g05 → g10 → g07 → g03	0.6 ± 1.4	0.7
g03 → g05 → g02 → g10 → g07 → g03	1.2 ± 1.5	1.2
g03 → g05 → g02 → g10 → g08 → g07 → g03	–1.5 ± 1.9	–1.4

### 4.3 Comparison with Experimental RBFE

**Figure 7:**
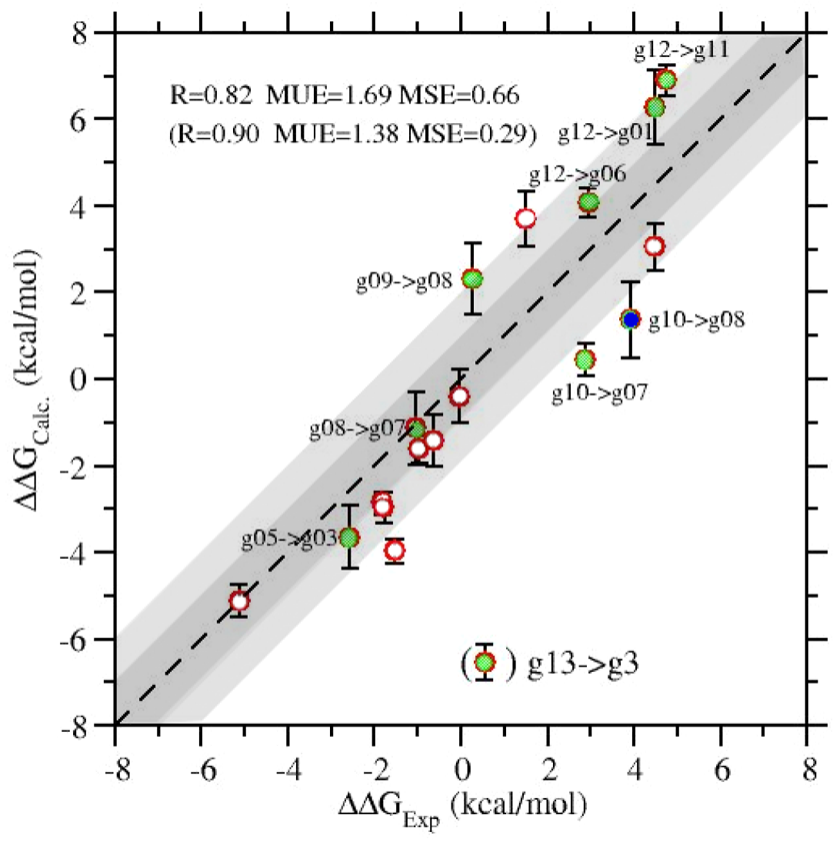
Correlation plot between the experimental^41^ and calculated RBFE of Table 2. White, green, and blue symbols indicate a charge change in the tansmutation of 0, 1, and 2 electrons, respectively.

In Figure 7, we show the correlation plot between the 18 experimental^41^ and calculated RBFE using NE-Alchemy. The latter include the finite-size correction δ*G*_fs_ reported in Table 2. Correlation is very good with a Pearson correlation coefficient of 0.82 and an MUE of 1.7 kcal/mol. In ref (47), we showed that the systematic overestimation of the absolute dissociation free energies of 2–3 kcal/mol was very likely, because of the large (+12 electrons) neutralizing background uniform charge distribution artificially causing the charged guest to favor the lower dielectric environment^66^ (i.e., the WP6 cavity).

For RBFEs, this systematic error largely cancels out, yielding an MSE of only 0.66 kcal/mol. Figure 7 also shows that the only *clear* outlier involves g13 (Paraquat). The latter molecule was also an outlier in our SAMPL7 submission^36^ for the cucurbituril-like open cavitand (CB-clip^67^) and was an outlier in our SAMPL9 submission,^47^ once the absolute dissociation free energies were down-shifted by the systematic bias, because of the background plasma. As discussed in ref (36), the positive charge distribution delocalized on the aromatic rings of g13 (a peculiarity of this molecule, with respect to all other guests, both in CB-clip SAMPL7 and WP6 SAMPL9) is very likely systematically polarized by the electron-withdrawing groups decorating the rims of the WP6 or CB-clip hosts in the bound state, an effect that cannot be captured by a nonpolarizable FF such as ours. If we eliminate the outlier g13 → g03 from the set, the correlation between experimental and calculated RBFE becomes excellent (*R* = 0.90) with MUE and MSE dropping to 1.38 and 0.29 kcal/mol, respectively.

**Figure 8:**
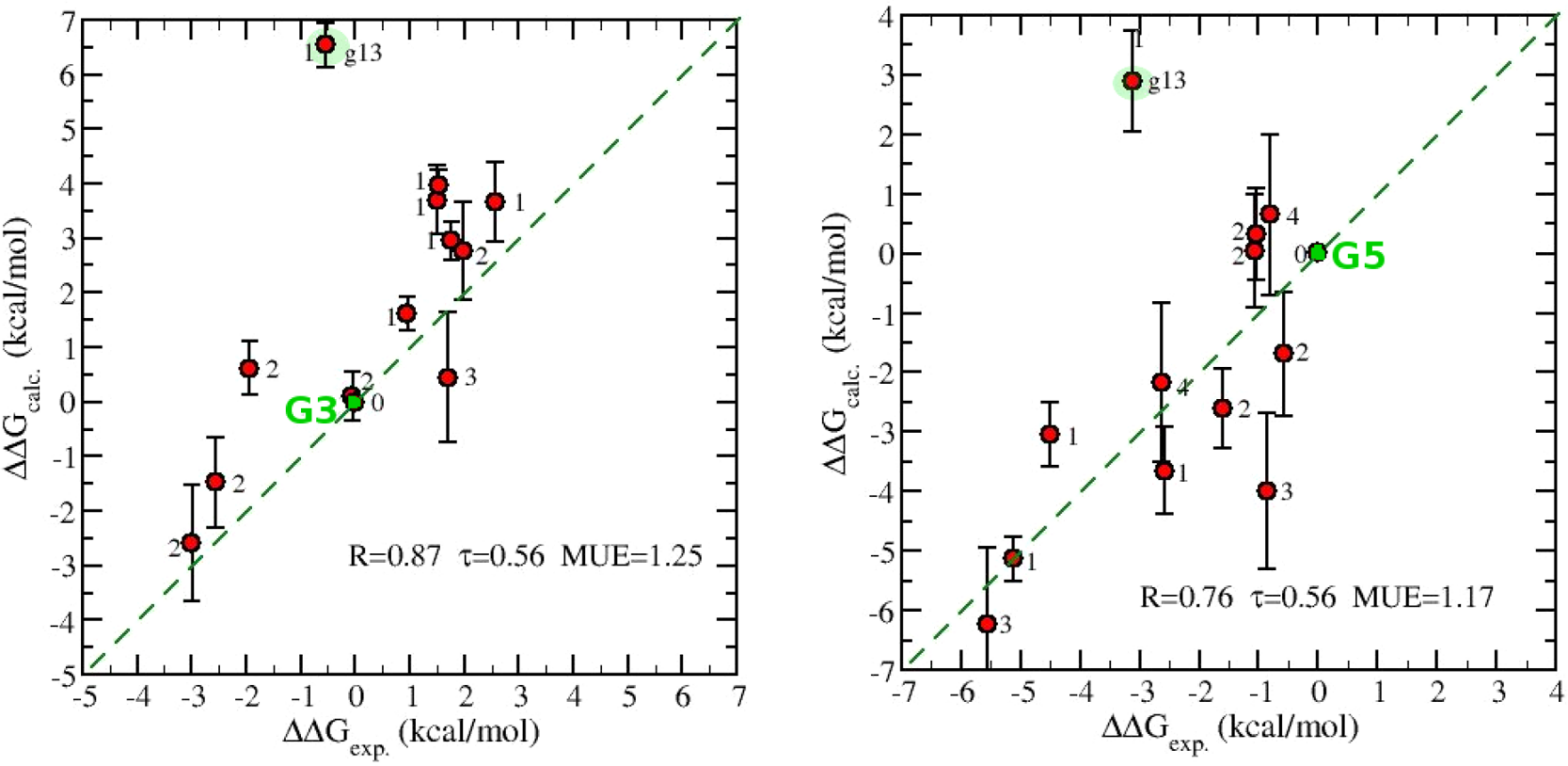
Calculated and experimental RBFE using G3 (left) and G5 (right) as references. *R*, MUE, and MSE have been computed by discarding the G13 outlier and the reference (see text). The number flanking the symbol represents the corresponding number of transmutations (shortest path) in the link (see Figure 2). The 12 connection paths for calculated RBFEs are reported in the SI.

In Figure 8, we finally report the comparison between experimental and calculated RBFE, taking g03 and g05 as references. These two guests are characterized by a strong chemical dissimilarity, with respect to volume, net charge, Tanimoto coefficient, and number of cycles. It should be stressed that most of the RBFEs refer, in this case, to *indirect* calculations involving two or more transmutations. For example, to arrive at g09, taking g03 as a reference, we have combined the results of four RBFEs as reported in the SI. The number flanking the symbols in the plot refers to the number of transmutations used in the final estimate (*N*_*t*_). Note that the error bars in the calculations are generally higher the larger *N*_*t*_ is, as the final confidence interval is obtained by summing in quadrature the *N*_*t*_ errors. Remarkably, the agreement with experimental data is not significantly dependent on the reference choices, despite their chemical dissimilarity.

## 5 Conclusion

NE-RBFE, implemented via a straightforward dual topology bidirectional scheme, has been shown to provide accurate (BAR-based) results in the challenging SAMPL9 test bed, irrespective of the chemical distance between the two compounds. Cycle closure conditions involving compounds differing in charge, volume, and Tanimoto coefficient are satisfied within the confidence interval, irrespective of the number of edges in the cycle. These two remarkable features are not within reach of standard ST FEP-based RBFE calculation. Besides, according to ref (64), a ST-FEP-RBFE calculation between two strictly congeneric compounds requires, on per edge basis, a total of ∼500 ns in the bound state (3 ns on each of the 20 λ-states and 10 replicates for assessing the confidence intervals requiring 0.5 GB of disk space of storage for a post-processing BAR-based calculation. NE-RBFE requires, on per edge basis, <300 ns in total in the bound state, storing only the final work data (<2 kB) for later analysis. In NE-RBFE, there is no need of of introducing restrained dummy atoms, as done in ST-FEP-RBFE. As recently shown,^24^ an inattentive choice of these restraints, by coupling the ligand–ligand internal coordinates, may produce a spurious contribution to the RBFE. The restraint in our approach is only between the COM of the two ligands with no impact, by design, on the internal degrees of freedom and on the final transmutation free energy as their cost cancels out at the end-states of the transformation. We have seen that NE-alchemy unidirectional estimates are accurate if the volume of the two compounds do not change much, even if the Tanimoto coefficient is well below 0.5. This gives the opportunity to avoid the HREM sampling on the arrival end-state and to cut the cost of NE-RBFE in half for nearly equal volume ligands, hence considerably accelerating hit-to-lead projects in drug design.

In standard FEP-RBFE, slowly intercalating water molecules during the transition are a well-known limiting factor in convergence.^68^ In NE-RBFE, this is no longer an issue, since the distribution of water molecules must be at equilibrium only at the end-states, which may be effectively sampled using efficient HREM schemes. In NE-RBFE, the alchemical path is crossed at fast speed and there is *no need* to canonically sample intermediate λ-states as in FEP-RBFE. Besides, in the dual topology NE-alchemical trajectories, thanks to the shifted potential of eq 3 and to the COM–COM tethering potential, the ligand region is kept constantly devoid of water molecule if the two compounds have approximately the same volume and shape. When the volume or shape is different, water molecules may enter or not into the region made available by the annihilating partner, but this may have an impact *on the dissipation* observed in the final work distributions, largely tamed by the intrinsic accuracy of the vDSSB approach in the unidirectional estimates or by the intrinsic accuracy of the BAR (vDSSB-boosted or not) bidirectional approach, even when the overlap of the work distribution is negligible.

We conclude by stressing that NE-RBFE can be implemented with little or no code modification in all popular MD programs that support HREM and NE alchemical transitions such as Gromacs, NAMD, or Amber. By affording the calculation of the RBFE for chemically distant (or scaffold-hopping-related) compounds in a matter of few hours on an HPC facility, NE-RBFE has the potential to become a powerful tool in the computer-based hit-to-lead drug discovery pipeline in industrial settings.

## Data and Software Availability

PDB trajectory files, raw work data, and force field parameter files are available at the general-purpose open-access repository Zenodo (https://zenodo.org/record/6385017).

The ORAC program (v6.1) is available for download under the GPL at the Web site http://www1.chim.unifi.it/orac/.

Third-party software Autodock Vina can be downloaded from the Web site https://vina.scripps.edu/.

## Appendix: Impact of COM-COM Restraints on the Alchemical Free Energy

Here, we show that the free-energy change of the alchemical transformation from the bound state A(B) to the bound state B(A) is formally independent of the COM–COM restraint potential between the two guests, if and only if such potential is *symmetrical*,^32^ with respect to the exchange of the A and B labels. In the absence of COM–COM restraint, the free-energy change of transforming A into B *in the bound state* is given by

5

Δ*G*_bound_ represents the reversible work to bring the bound guest A into the gas phase while simultaneously bringing the B guest from the gas phase to the bound state. The label in parentheses indicates that the corresponding molecule is decoupled from the environment, and *Z*_A(B)_ and *Z*_B(A)_ are the configurational partition functions of the two thermodynamic end-states {A(B) _b_, {B(A)}_b_. Their potential energy is given by

6

where **x**_A_, **x**_B_, **x**_H_ denotes the *internal* coordinates of the guests and the host, **r**_s_ are the coordinates of the solvent and ζ_A_, ζ_B_ are the host–guest external coordinates. The latter are six positional and orientational coordinates ζ ≡ **R**, ω where **R** is the host–guest COM-COM distance and ω are the three euler angles defining the relative host–guest orientation.^69^*U* and *U*_0_ in eq 6 refer the host–guest potential energy and the intramolecular potential energy of the guest, respectively. Note that the potential energy *V* is independent of the host–guest external coordinates of the decoupled molecule. Using eq 6 in eq 7, we have

7

where *I*(ζ_A_), *I*(ζ_B_) are the so-called indicator functions of the relative host–guest external coordinates marking the region of existence of the HA and HB complexes. *I*(ζ) is equal to one if the host–guest relative coordinates ζ correspond to a bound configuration, and zero otherwise.^32,69−71^ Note that, in the second equality of eq 7, we have integrated away the decoupled ζ_B_ and ζ_A_ coordinates in the numerator and denominator, respectively, obtaining, in both cases, an 8π^2^*V* term, which simplifies.

In presence of the position (COM–COM) restraint, the potential energy (eq 6) includes the additional term *u*_*r*_(ζ_AB_):

8

where the symmetrical *u*_*r*_(ζ_AB_) potential is equivalent to the COM–COM harmonic potential used in the NE alchmical tranformations, i.e.,

9

Here, the restraint potential is dependent only on the *positional* host–guest **R** external cordinates and is symmetrical with respect to the exchange of the labels. It may also involve orientational ω coordinates as well, as long as *u*_*r*_ remains symmetrical, with respect to the exchange of the label.^32^ Using eq 8, we find that, for the restrained system, the free-energy change is given by

10

where the integration domain of the ζ_AB_ coordinates is the volume of the system and entire solid angle:

11

The two identical integrals, depending on the ζ_AB_ coordinates on the denominator and numerator of eq 10, simplify, recovering eq 7 hence showing that Δ*G*_bound_^′^ = Δ*G*_bound_, QED. We stress once more that the free energy estimate (eq 10) is independent of the COM–COM restraint potential if and only if the latter is symmetrical, with respect to the exchange of the guest labels.

## Supporting Information Available

Bound and unbound state work distributions for all 18 forward and reverse transmutations of Table 2 (Figure S1–S18); dissipation in RBFE and ABFE calculations as measured by the variance of the work distributions (Figure S19) (PDF)
