# Supplementary material for: Relative Binding Free Energy between Chemically Distant Compounds Using a Bidirectional Nonequilibrium Approach
Source: J Chem Theory Comput. 2022 Jun 1;18(6):4014–26. doi: 10.1021/acs.jctc.2c00295 (PMC9202353; doi:10.1021/acs.jctc.2c00295)
Supplement: Supplementary file 1 — ct2c00295_si_001.pdf [file ct2c00295_si_001.pdf]

Supporting Information for "Relative binding free energies between chemically distant compounds using a bidirectional nonequilibrium approach"

Piero Procacci*

Dipartimento di Chimica "Ugo Schiff", Universit degli Studi di Firenze, Via della Lastruccia 3, Sesto Fiorentino, I-50019 Italy

E-mail: procacci@unifi.it

Forward vDSSB process: black curves refer to the work distribution $P_b * P_u(W)$ given by the convolution of the alchemical work due to trasmutation of A into B in the bound state ($P_b(W_{A->B})$, with the alchemical work due to trasmutation of B into A in the unbound state ($P_u(W_{B->A})$).

Reverse vDSSB process: Red curves refer to the work distribution given by the convolution $P_b * P_u(-W)$ of the alchemical work due to trasmutation of B into A in the bound state ($P_b(W_{B->A})$), with the alchemical work due to trasmutation of B into A in the unbound state ($P_u(W_{A->B})$).

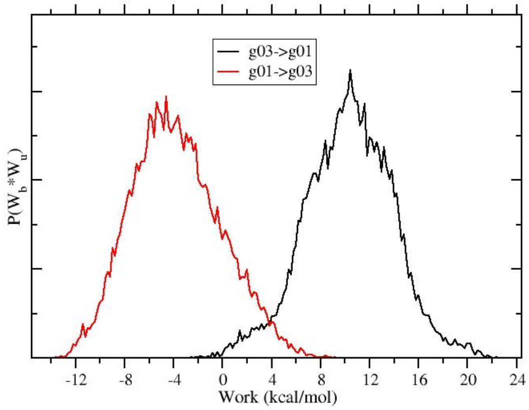

Figure S1

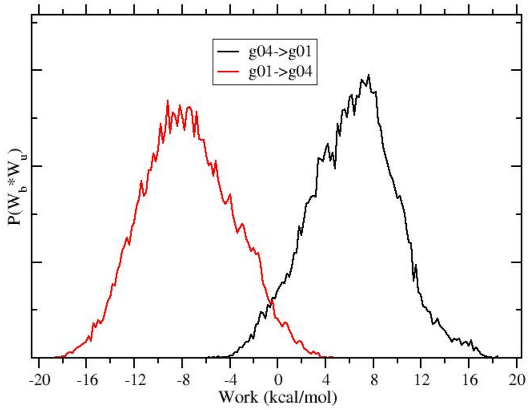

Figure S2

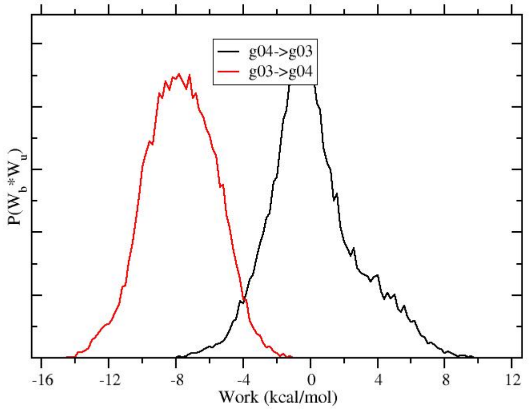

Figure S3

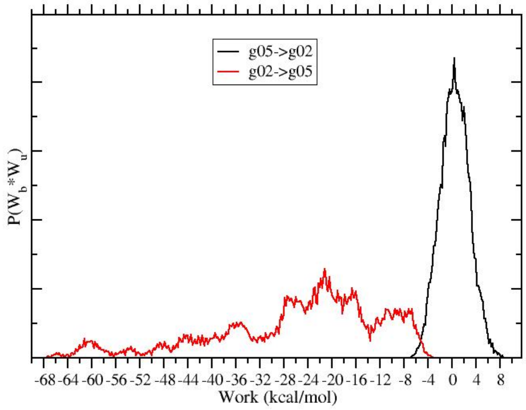

Figure S4

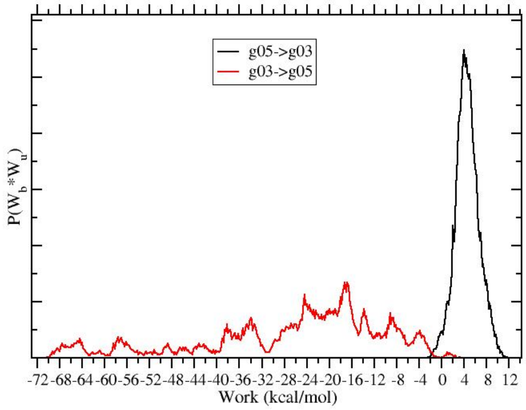

Figure S5

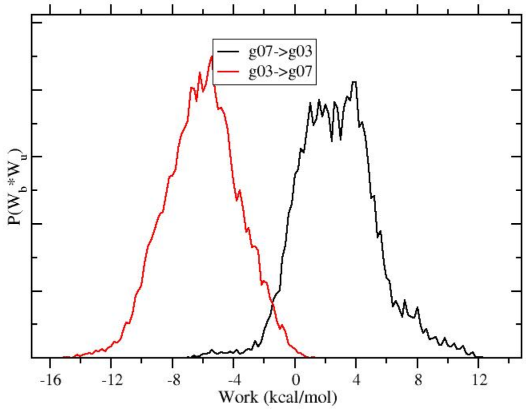

Figure S6

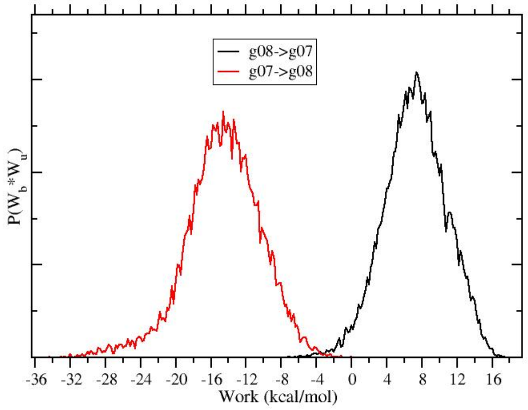

Figure S7

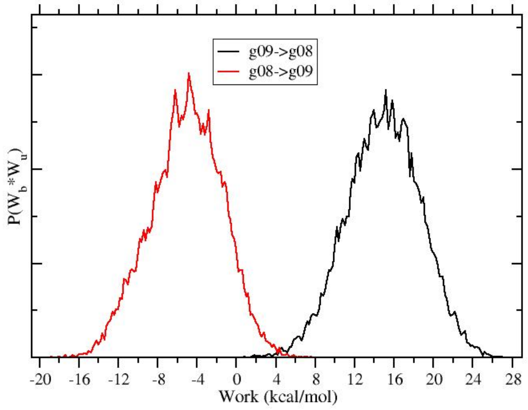

Figure S8

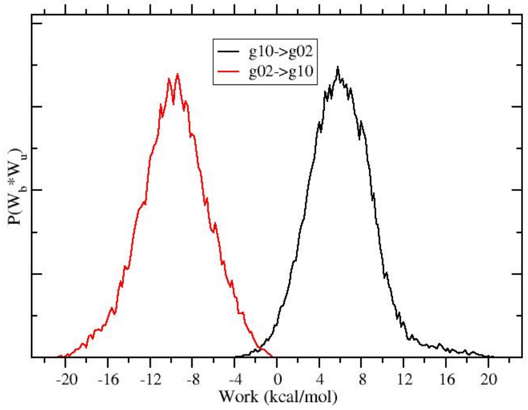

Figure S9

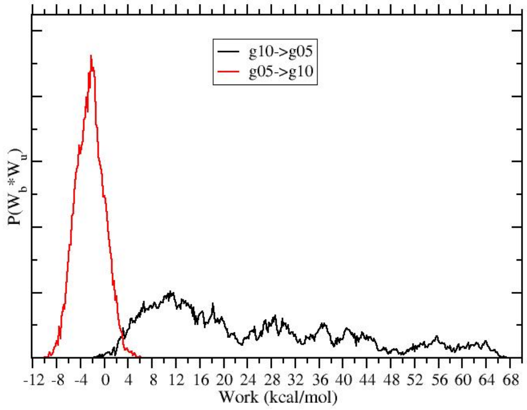

Figure S10

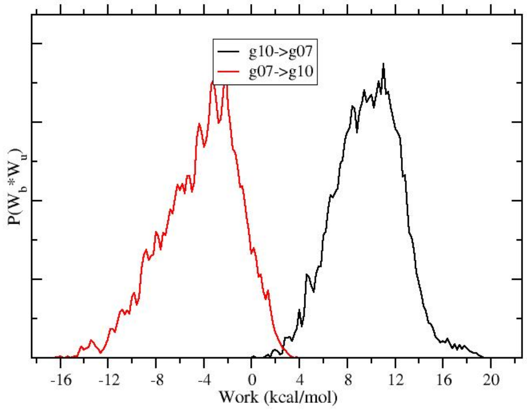

Figure S11

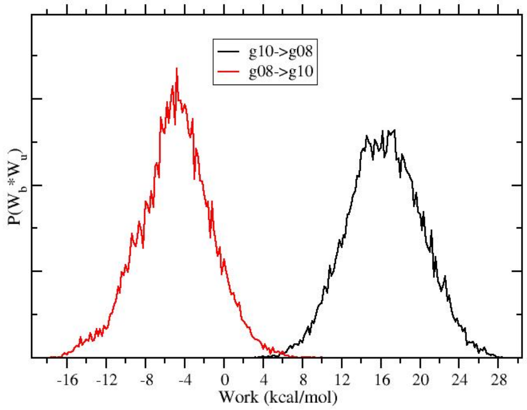

Figure S12

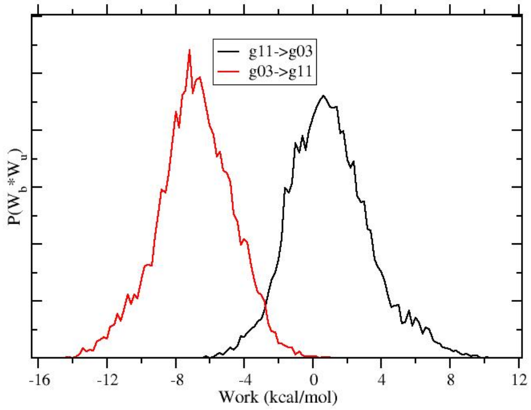

Figure S13

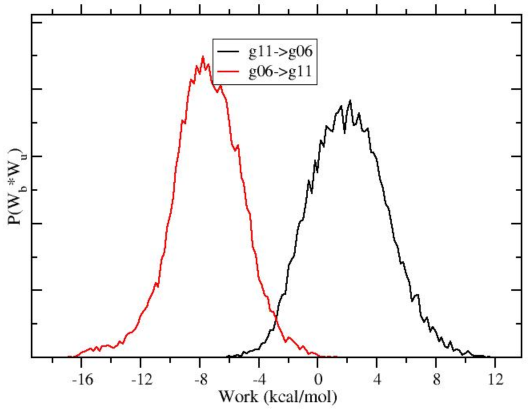

Figure S14

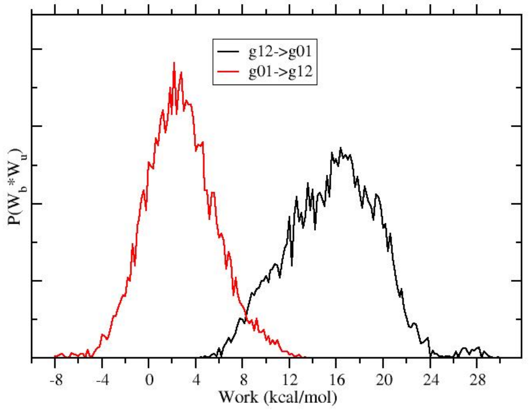

Figure S15

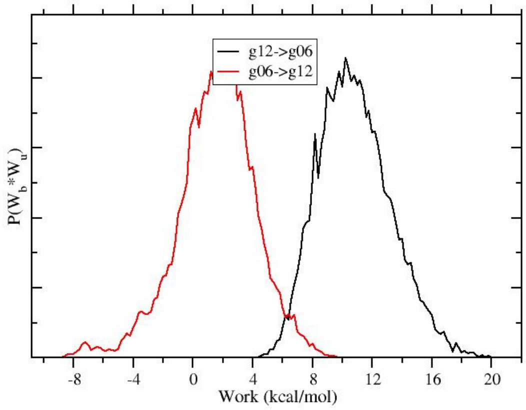

Figure S16

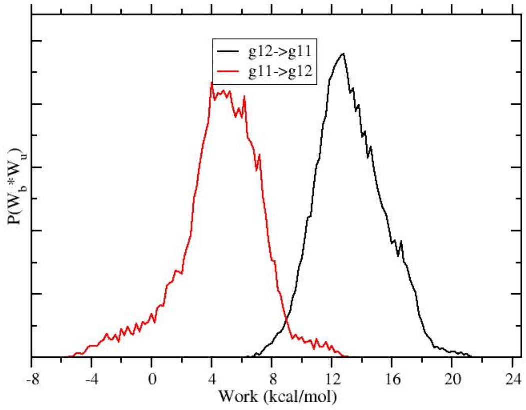

Figure S17

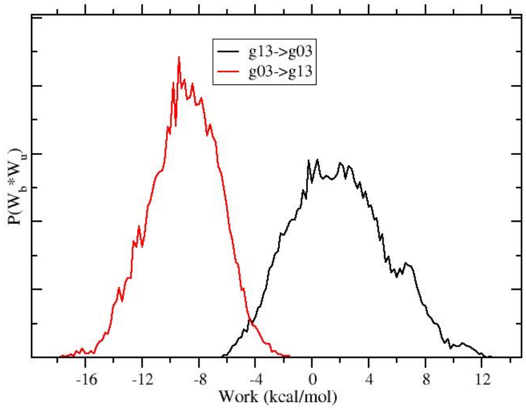

Figure S18

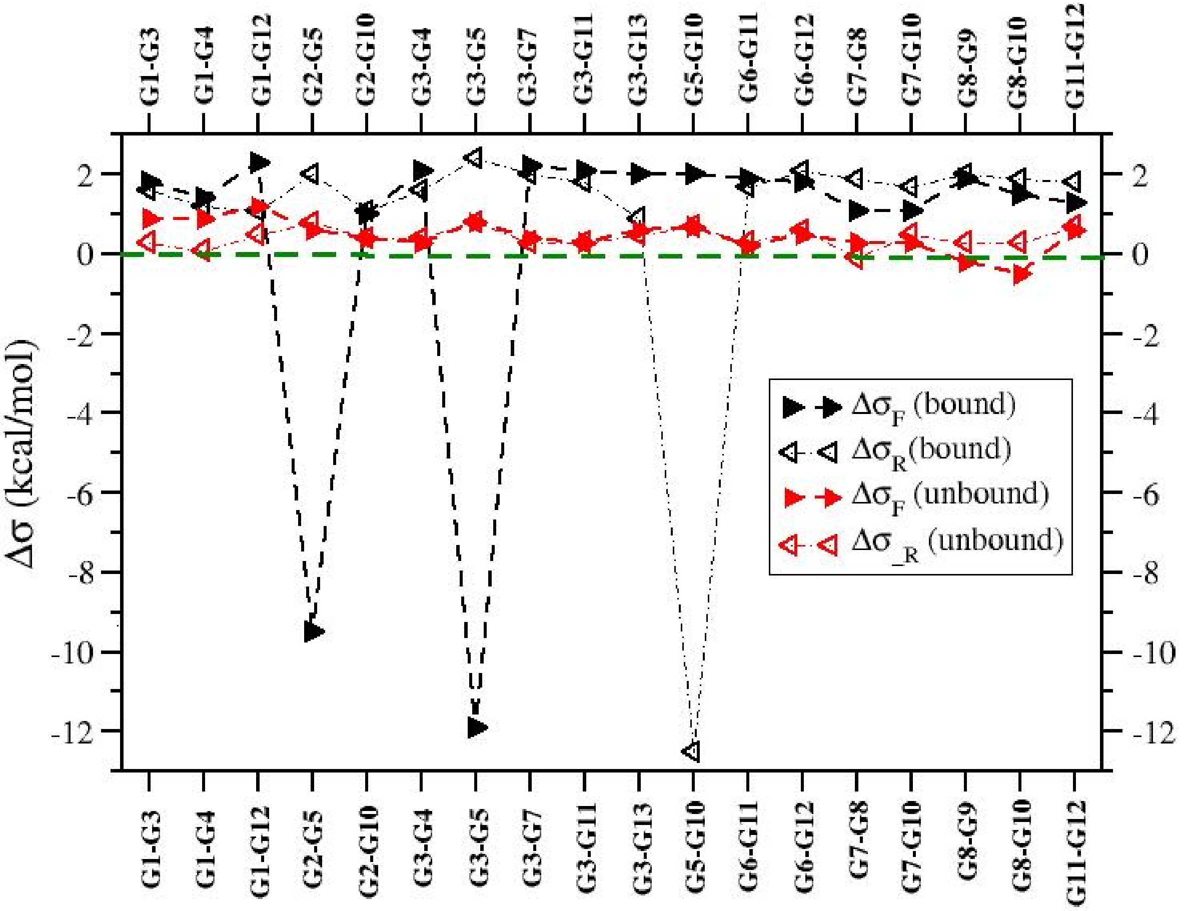

Figure S19: $\Delta\sigma = \sigma_{\text{ABFE}} - \sigma_{\text{RBFE}}$ is the difference in the standard deviation of the work distribution (related to the dissipation) between two ABFE and one RBFE calculation For the two ABFE calculation, the total variance for the work $W = W_g + W_a$ is given by $\sigma^2_{\text{ABFE}} = \sigma^2_g + \sigma^2_a$, where the subscripts g, a mark the growth and annihilation process. The variance of the RBFE DT process is always less than the (theoretical) variance σ_{ABFE} for the unbound state. For the unbound state, σ_{RBFE} is much larger than σ_{ABFE} only for those processes where the bulky g05 starts as a ghost compound.

Paths with G3 as reference [Figure 8 (left) of the main paper]

```
#P g03 g01

#P g03 g04

#P g03 g05

#P g03 g13
```

```
#P g03 g07

#P g03 g11

#P g03 g01 g12

#P g03 g11 g06

#P g03 g05 g02

#P g03 g05 g10

#P g03 g07 g08

#P g03 g07 g08 g09
```

Paths with G5 as reference [Figure 8 (right) of the main paper]

```
#P g05 g03

#P g05 g02

#P g05 g10

#P g05 g10 g07

#P g05 g10 g08

#P g05 g10 g08 g09

#P g05 g03 g01

#P g05 g03 g04

#P g05 g03 g13

#P g05 g03 g01 g12

#P g05 g03 g01 g12 g11

#P g05 g03 g01 g12 g06
```
